# Evaluation of PIRs Post-Fire Pull-Out Strength in Concrete Exposed to ISO 834-1 Fire

**DOI:** 10.3390/ma14174998

**Published:** 2021-09-01

**Authors:** Nagham Abdelrahman Alhajj Chehade, Amine Lahouar, Omar Al-Mansouri, Nicolas Pinoteau, Marco Abate, Sébastien Remond, Dashnor Hoxha

**Affiliations:** 1 Centre Scientifique et Technique du Bâtiment (CSTB), 84 Avenue Jean Jaurès, Champs-sur-Marne, CEDEX 2, 77447 Marne-la-Vallée, France; lahouaramin@hotmail.com (A.L.); Omar.ALMANSOURI@cstb.fr (O.A.-M.); Nicolas.PINOTEAU@cstb.fr (N.P.); 2 Laboratoire de Mécanique Gabriel Lamé (LaMé), Université d’Orléans, 45100 Orléans, France; sebastien.remond@univ-orleans.fr (S.R.); dashnor.hoxha@univ-orleans.fr (D.H.); 3 Hilti Corp., 9494 Schaan, Liechtenstein; Marco.Abate@hilti.com

**Keywords:** post-fire, concrete, post installed rebars, pull-out tests, numerical model, bond strength, resistance integration method

## Abstract

Post-installed rebars (PIRs) using mortar can offer bond strength at ambient temperature equal or higher to that of cast-in place rebars. However, high temperatures have the effect of weakening the bond, typically governed by the chemical and physical properties of the mortar which is often sensitive to temperature increase. Therefore, the behavior of PIRs in a fire situation becomes vulnerable. Moreover, after exposure of PIRs to high temperature, the heat transfer continues during the post-fire phase, which might endanger the construction after a fire event. In order to evaluate the evolution of the pull-out capacity during fire, Pinoteau et al. have developed the bond resistance integration method (Pinoteau’s RIM) to predict the bond resistance value of a rebar subjected to various temperatures in accordance with the fire exposure curves. Therefore, accurate temperature profiles during the post-fire phase are needed to ensure a correct calculation of the post-fire behavior of the PIR connection. This paper presents 3D finite element thermal simulations of PIRs in concrete exposed to ISO 834-1 fire conditions then cooled with ambient air. Numerical thermal profiles are then compared to the experimental results (i.e., post-fire pull-out tests). The proposed model provides guidelines for conducting numerical simulations to determine the thermal entry data necessary for predicting thermal profiles in PIRs during heating and cooling phases. Then, the post-fire pull-out capacity of PIRs in concrete is calculated using Pinoteau’s RIM, and compared to experimental post-fire pull-out results.

## 1. Introduction

In recent years, there has been a growing interest in using anchoring systems in reinforced concrete structures. Thus, anchorage systems for reinforcement applications are classified into two main categories: “cast-in place rebars” and “post-installed rebars (PIRs)”. PIRs are very popular in the strengthening of concrete structures in construction projects. They offer a viable method for adding new concrete sections to existing structures [1] and provide the flexibility in the construction sequence. According to the bonding agents typically used for PIR applications, PIRs can be divided into two groups [2]: “polymeric PIRs” where the bonding agent is organic such as epoxy, polyester, and vinylester mortars, and “grouted rebars” where the bonding agent is cementitious. Post-installed rebars may fail in different failure modes under tensile loading: concrete cone failure in an unconfined tension setup, steel failure, pull-out failure, and splitting failure.

The mechanical properties of PIRs made with mortar are governed by several parameters such as materials, geometry, and installation procedure [3,4,5]. PIRs have high mechanical properties at ambient temperature, however, for the majority of the mortars used in PIRs, when exposed to high temperatures, the bond resistance decreases. The material properties undergo major changes, particularly, the material strength and stiffness change with increasing temperatures. Lahouar et al. [6] showed that the mechanical properties of PIRs made with polymeric mortars improve when the bond temperature rises up to values below the mortar glass transition temperature due to the post-cure phenomenon. However, when temperature exceeds the mortar glass transition temperature, a change in the viscoelastic properties of the polymer occurs, which results in creep and high sensitivity to temperature [7,8]. Pinoteau [9] studied the influence of the increase of heating rate on bond failure of post-installed rebars made with mortar in concrete using polymeric mortars, and concluded that a bond stress redistribution phenomenon occurs due to the thermal gradient along the steel rebar. Previous pull-out tests at high temperature have shown that bond capacity decreases rapidly with temperature [6,9]. Al-Mansouri et al. [10,11] have studied the influence of fire exposure conditions on the pull-out resistance of bonded anchors in concrete. Pinoteau et al. [12] proposed a “bond Resistance Integration Method” using thermal calculations and bond resistance variation at different temperatures. This design method at high temperature was developed allowing to predict the pull-out resistance of post-installed rebars [12]. Pinoteau’s method was validated by performing a full-scale ISO fire on a cantilever-wall connection using PIRs made with polymeric mortars [12]. Lahouar et al. also proposed a non-linear shear lag model taking into account the displacement compatibility between PIRs elements when exposed to high temperatures in the design process (Lahouar’s shear-lag model) [13]. Both approaches yielded conservative predictions of fire resistances. However, scarce are the studies that have been carried out to establish the behavior of PIRs during the post-fire.

This paper presents first the experimental study to investigate the post-fire bond behavior of PIRs made with polymeric mortars. Then, the thermal behavior in the experimental program is modeled using a transient heat transfer analysis via Ansys. The aim was obtaining numerically the temperature profiles along PIRs in concrete exposed to ISO 834 fire followed by air cooling. Using this input data of temperature with the bond stress vs. temperature relationship for the used mortar, the post-fire pull-out capacity of the PIR is calculated by numerically integrating the temperature-dependent bond strength along the embedment depth of the PIR. The results of the predicted post-fire capacity were compared to experimental test results. This paper shows the comparison between the experimental and numerical temperature profiles along the PIR during both fire and post-fire phase. It also shows the comparison between the calculated post-fire pull-out capacities with respect to the experimentally obtained post-fire pull-out test results. The calculated thermal profiles of the 3D transient thermal analysis and the bond strength temperature relationship of the used mortar in the experimental program were used to predict the post-fire pull-out capacities using Pinoteau’s method. Results have shown that the 3D model yields conservative calculations of the temperature profiles in both fire and post-fire phases, which consequently led to conservative predictions of the post-fire resistance.

## 2. Materials and Experimental Procedure

The post-fire tests aimed to investigate the bond behavior of PIRs made with polymeric mortars during the post-fire phase [14] and to provide a database for a post-fire design method, based on Pinoteau’s method [12]. The experimental protocol was divided into two parts: fire tests were carried out for exposure of 90 min on post-installed rebars made with resin-based mortars. Then, post-fire pull-out tests were conducted at several cooling times with a confined test setup displacement controlled tests [15].

### 2.1. Description of Tested Materials

Fire tests were carried out on 6 reinforced uncracked concrete beams of 300 mm of width, 1200 mm of length, and 400 mm of thickness. The fire tests were conducted on post installed rebars of 16 mm diameter (GEWI type), using an epoxy-based mortar. The PIRs were anchored in concrete with 155 mm (±3 mm) embedment depth. The embedment was chosen as 10 φ [15], where φ is the diameter of the rebar. It was chosen to ensure a pull-out failure with the highest possible thermal gradient. The holes were drilled to the required embedment depth with a hammer drill set in rotation-hammer mode with a medium size cutting diameter $d_{cut,m}$. The composition of the concrete used for the manufacturing of beams is presented in Table 1. Characterization tests were carried out on cubic concrete samples (150 mm × 150 mm × 150 mm) after 28 days of curing under ambient temperature and moisture conditions. The concrete used for casting was a C20/25, it showed a compressive strength equal to 25.8 MPa and a density equal to 2207 kg/m^3^.

The ultimate tensile stress value and the yield stress value of the rebars were also determined (*f_u,test_* = 670 MPa, *f_y,test_* = 577 MPa). The steel failure of the used GEWI rebar occurred at mean value of 135 kN based on a series of three tests.

### 2.2. Description of the Test Set-Up

**Figure 1:**
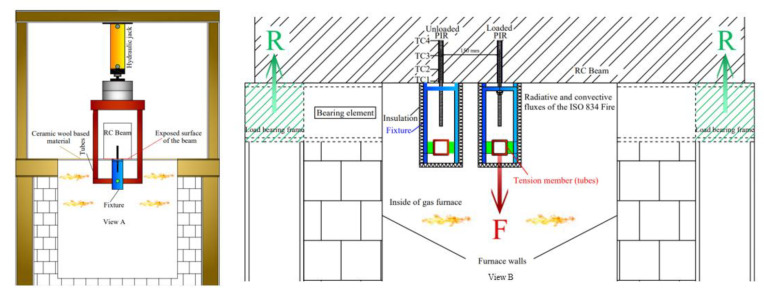
View of the gas furnace and the loading system during the heating phase.

**Figure 2:**
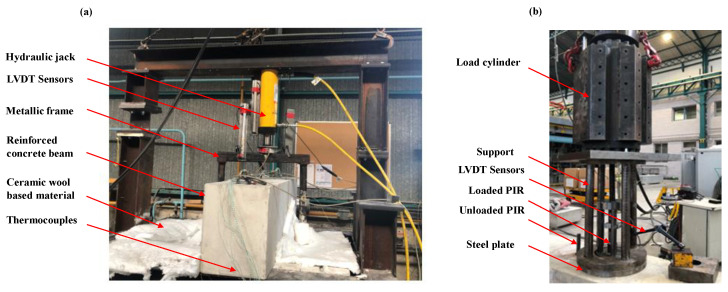
Testing apparatus in the heating phase (**a**) and the cooling phase (**b**).

Fire tests were conducted at the fire resistance laboratory at CSTB according to the specification of EOTA TR 020 [16]. Two PIRs were installed as anchors on one side of each reinforced concrete beam, one of which was mechanically loaded, while the other was equipped with type K thermocouples (presented by TC1 to TC4 in Figure 1) along the embedment depth to measure the temperature profiles of the PIR at the steel/mortar interface. The PIRs were anchored in the concrete beams with the epoxy-based mortar in beams B1 to B6 as in Table 2. The beam was then placed on top of a gas furnace with PIRs on the fire exposed surface. The fire exposed surface of the beam was 600 mm × 300 mm. The distance between the loaded PIR centered above the furnace and the unloaded PIR was equal to 150 mm. This aimed at replicating the same temperature profiles of the unloaded PIR in the loaded counterpart, where no thermocouples were positioned on the mechanically loaded PIR to avoid affecting the adherence between the mortar and the rebar. During exposure to fire conditions, the mechanically loaded PIRs were loaded in tension under a constant load corresponding to 4.5% of the mortar strength at ambient temperature. The load-transfer between the post-installed rebar and the concrete is achieved through bond and friction along the embedment depth. Linear variable differential transformer (LVDT) sensors were used to measure the rebar displacement as presented in Figure 2.

The loading system during the heating phase used hydraulic jacks, applying a downward mechanical load (presented by F in Figure 1, R being the reaction force) on a system of metallic frame surrounding the reinforced concrete beam. The metallic frame was connected to the fixture of the post installed rebar inside the furnace. Standard metallic fixtures for such fire tests were used [16]. The influence of the loading systems on temperature profiles and resulting pull-out capacity of bonded anchors in the heating phase were investigated by [10,11] and found negligible. The steel of the loading system was insulated using a ceramic wool-based material to protect the steel from reaching failure and to induce a pull-out (bond) failure. The same thermal boundary conditions were applied on both loaded and unloaded PIR.

Afterward, the test specimens were exposed to 90 min of heating following the ISO 834-1 fire curve inside the gas furnace. The heating time was chosen in a way that the maximum temperatures along the bond would be lower than 300 °C. This temperature was selected because most mortars typically provide bond strengths lower than 1 MPa beyond this value according to characterization tests [15]. In order to obtain a post-fire pull-out failure and to prevent steel failure during the tests, this maximum temperature at the steel/mortar interface was considered in the design procedure to guarantee a pull-out failure during the cooling phase. Afterwards, the test specimen was removed from the oven and left to cool down for several cooling times as presented in Table 2.

The next step consisted of conducting the post-fire pull-out tests at the different cooling times by applying a tensile force. The surface of concrete was confined in order to prevent concrete cone failure. The applied tensile load was displacement controlled. Figure 2a shows the testing apparatus during heating, where the reinforced concrete beam was placed on the gas furnace. Figure 2b shows the testing apparatus (confined test setup) after fire exposure in the cooling phase.

## 3. Experimental Results

**Figure 3:**
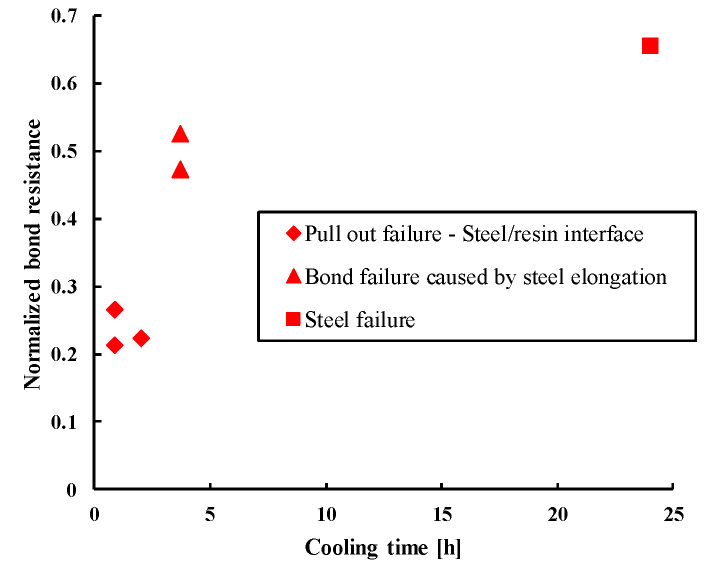
Post-fire pull-out normalized resistances at different cooling times.

The results of post-fire pull-out tests according to the cooling time are shown in Figure 3. The experimentally obtained bond resistance values were normalized, where 1 corresponds to the reference bond resistance in uncracked concrete at ambient temperature.

The normalized resistance was calculated using the following equation:

(1) $$F_{normalized}=\frac{F_{experimental\;}}{F_{calculated\;at\;ambient\;temperature}}$$

(2) $$F_{calculated\;at\;ambient\;temperature\;}=\tau_{ambient\;temperature}\times \pi \times d\times l$$

where:

$\tau_{ambient\;temperature}$ is the mean bond resistance at ambient temperature (based on 3 reference confined tension tests according to EAD 330087 [15]).

d is the diameter of the rebar.

l is the embedment depth of the rebar.

Results suggest that the pull-out capacity of the tested epoxy-based mortar increases with cooling time, indicating a strength recovery of this mortar during the cooling phase. It is pointed out that the failure mode obtained during post fire tests was pull-out failure at the steel/mortar interface, except for cooling times of 3 h:40 min and 24 h, where bond failure caused by steel elongation for the former and steel failure for the latter case were observed. For the cooling time of 24 h, the steel failure obtained with the chosen embedment depth of 10 φ highlights the strength recovery of this mortar during the cooling phase.

The duration of cooling phase before testing (up to 24 h) is chosen in accord with the aim of this study to determine the post-fire bond resistance during the transitional phase, immediately after the fire is stopped and before the full cooling. In fact, it is thought and we checked it experimentally (see Figure 3) that there is a recovery (at least partially) of bond strength after this transitional period. Because of this recovery of strength when the temperature decreases, one intuitively could think that the strength during the transitional cooling phase would be a monotonically increasing function of the cooling time, with the lowest value immediately. In fact, during this transitional phase the temperature along the bond continued to rise, even if the fire is stopped, which could lead to a bond strength lower than the strength at the end of the fire.

The results of experimentally obtained temperatures for all the beams mentioned in Table 2, and the load vs. displacement curves for the epoxy-based mortar are detailed in [14].

## 4. 3D Transient Thermal Analysis Using Ansys

This section describes the transient heat transfer analysis used for predicting the temperature profiles along the PIR. Predicted temperatures are then subjected to Pinoteau’s method for the determination of post-fire pull-out capacity. The temperatures at any given time of fire exposure followed by cooling are calculated using a 3D transient thermal analysis performed on Ansys. Concrete and steel components properties were taken into account in the model (modeled as cast-in rebar), but not the mortar layer of 2 mm around the PIR since its influence on thermal profiles is negligible [17].

### 4.1. Description of the 3D Transient Thermal Analysis

To predict the temperature distribution in the system during fire exposure followed by cooling, the transient heat transfer analysis was divided into three steps. The objective of these steps was to simulate the following phases in the experimental procedure. The first step consists of heating the specimen for 90 min following the ISO 834-1 fire curve. Then, the second step consists of removing the beam from the furnace and removing the fixture and insulating materials and lasted 25 min. Finally, the third step consists of cooling the system to ambient temperature. Carbon steel and concrete material properties injected in the model are given by the Eurocode 2, part 1–2 [18]. In the numerical model, the concrete remains uncracked and concrete spalling was ignored. Concrete and steel were modelled as solid elements in Ansys. The connection between steel and concrete is a bonded interface (i.e., full thermal contact between concrete and steel). This allows no sliding or separation between edges, resulting in perfect contact between the inner surface of the hole in the concrete and the outer surface of the rebar.

**Figure 4:**
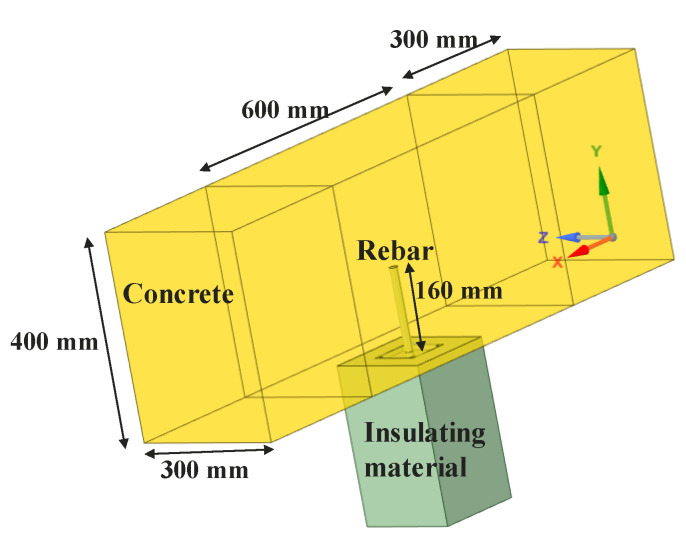
Geometry of the 3D modelling in Ansys.

The 3D model considers the PIR as a perfect cylinder inside the concrete beam with an embedment depth of 160 mm of the steel inside the concrete, and 40 mm of extended length (Figure 4). Al Mansouri et al. [11] conducted a parametric study to investigate the influence of the extended length of the steel above the concrete surface and concluded that this external length influences temperature profiles between no extended length and 20 mm of extended length. Beyond 20 mm from the concrete surface, the influence is insignificant for a medium size anchor (M12). It was also concluded that insulated fixtures significantly reduce the temperature profile along the anchors exposed to fire conditions compared to uninsulated fixtures.

**Figure 5:**
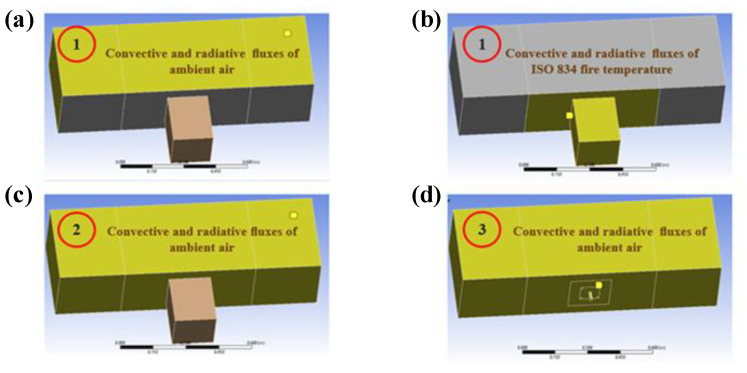
Boundary conditions applied in the three steps of the transient thermal analysis. To sum up, subfigures (**a**,**b**) in Figure 5 represent boundary conditions for step 1, subfigure (**c**) for step 2, and subfigure (**d**) for step 3.

During the heating phase where the specimen was exposed to 90 min of heating following the ISO 834-1 fire curve, the heat transfer occurs on the exposed surface of concrete via convection and radiation modes. The heat propagates inside the specimen only via conduction mode. The three steps in the experimental procedure described earlier are translated by the modification of boundary conditions in each step of the 3D model. Consequently, the first step in the 3D model consists of applying convective and radiative fluxes conditions corresponding to the ISO 834 fire [18] and ambient air respectively on exposed and unexposed surface of the concrete elements. The next step consists of applying convective and radiative fluxes of ambient air to the concrete beam, and adiabatic (zero heat flux) boundary conditions to the insulating material for 25 min. Finally, the fixture and insulated materials were removed from the geometry of the 3D model, and convective and radiative fluxes of ambient air were applied to the system of the concrete beam and the rebar. Figure 5 resumes the boundary conditions applied to the geometry in the three steps of the transient heat transfer analysis, where each number on the figure corresponds to the step number. 

The heat fluxes applied on the exposed and unexposed surfaces have two components:

(3) $$\mathrm{Convective}\;\mathrm{fluxes}:\;q_{conv}=h\;\left(T_{external}-T_{surface}\right)$$

(4) $$\mathrm{Radiative}\;\mathrm{fluxes}:\;q_{rad}=\sigma \varepsilon \;\left(T_{external}^{4}-T_{surface}^{4}\right)$$

where:

h is the convective heat transfer coefficient $\left({W\;m}^{-2}\;{}^{\circ}C\right)$

σ is the Stefan-Boltzmann constant $\left(5.67\;10^{-8}\;{W\;m}^{-2}\;{}^{\circ}C^{-4}\right)$

ε is the emissivity of concrete and steel (0.7)

The emissivity of concrete and steel and the convective heat transfer coefficient for the fire exposed surface are given by the Eurocode 2, part 1–2 [18] and are presented in Table 3. The convective factor for the surface exposed to ambient air figures in EN 1991-1-2 [19].

**Figure 6:**
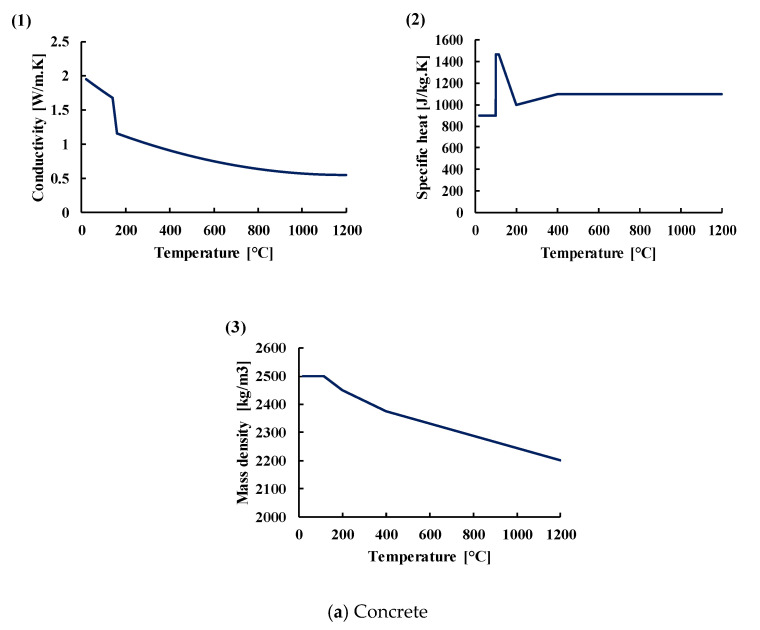

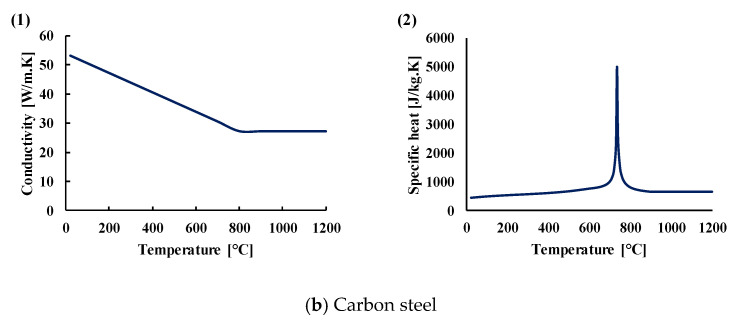
Variation of thermal properties of concrete and carbon steel. Subfigures (1) in subfigures (**a**,**b**) of Figure 6 represent the variation of conductivity with temperature for concrete and carbon steel respectively. Subfigures (2) in subfigures (**a**,**b**) of Figure 6 represent the variation of specific heat with temperature for concrete and carbon steel respectively. Subfigure (3) in subfigure (**a**) of Figure 6 represent the variation of mass density of concrete with temperature.

**Figure 7:**
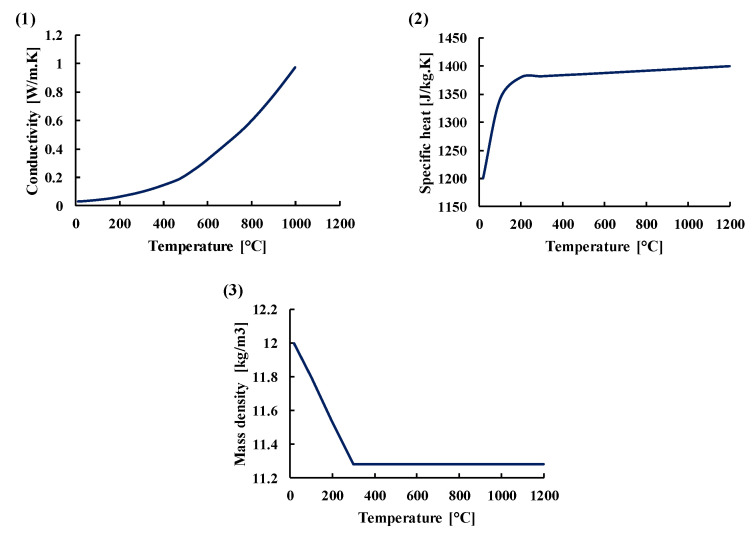
Variation of thermal properties of the insulating material used in the numerical analysis. The insulating material consisted of glass fiber with a thickness of 50 mm whose properties (conductivity, specific heat and mass density) are respectively presented in subfigures (1), (2), (3).

The thermophysical properties (conductivity, specific heat, and mass density) for both steel and concrete according to the French national annex in Eurocode 2, part 1–2 [18] are presented in Figure 6. The mass density of the carbon steel is considered constant equal to 7850 kg/m^3^ with respect to temperature. The thermal properties of the insulating material considered in the 3D numerical analysis were the same that were defined by Al Mansouri et al. [11]. The insulating material consisted of glass fiber with a thickness of 50 mm whose properties (conductivity, specific heat and mass density) are respectively presented in subfigures (1), (2), (3) of Figure 7. In fact, the insulating material in the experimental tests was a ceramic-based material and had better insulating properties. Therefore, using these properties for the insulating material in the 3D model leads to results of temperature profiles on the safe side.

The transient heat transfer analysis conducted via Ansys provided the temperature profiles injected in Pinoteau’s RIM. The bonded length was divided into 48 elements of 3.33 mm length, where temperature was considered uniform. The bond stress vs. temperature relationship for the tested mortar, which is the second input for the calculation of the load capacity of PIR, was obtained from tests according to [15].

The post-fire pull-out capacity of the PIR at any given time was calculated according to the following equation:

(5) $$N_{R,p}=\pi d\int_{0}^{h_{ef}}f_{bd,m}.k_{fi}\left(\theta \left(x\right)\right).dx$$

where: $N_{R,P}$ is the post-fire pull-out capacity (N).

d is the nominal diameter of the rebar (mm).

$f_{bd,m}$ is the mean bond resistance at ambient temperature (N/mm^2^).

$k_{fi}\left(\theta \left(x\right)\right)$ is a temperature-dependent reduction factor.

$\theta \left(x\right)$ is the temperature distribution along the embedment depth of the post-installed rebar.

$h_{ef}$ is the embedment depth of the rebar (mm).

### 4.2. Validation of the Model

To validate the numerical model, experiments from [14] and that described in Section 2.2 were used. For validating post-fire load calculations, Pinoteau’s RIM was applied using both temperature profiles predicted from the numerical model, and experimental temperature profiles. The results were then compared to the experimental post-fire pull-out resistances.

**Figure 8:**
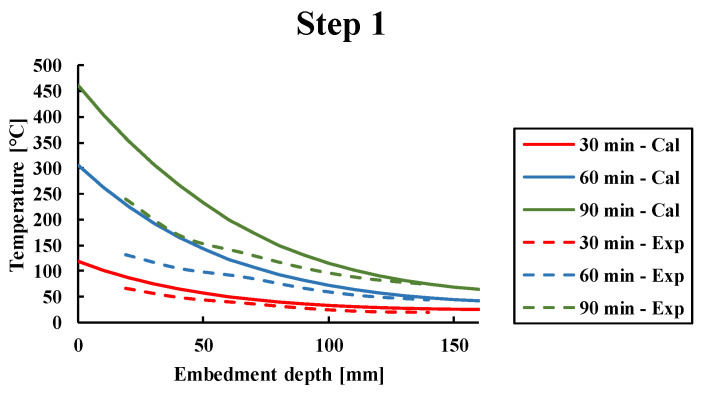

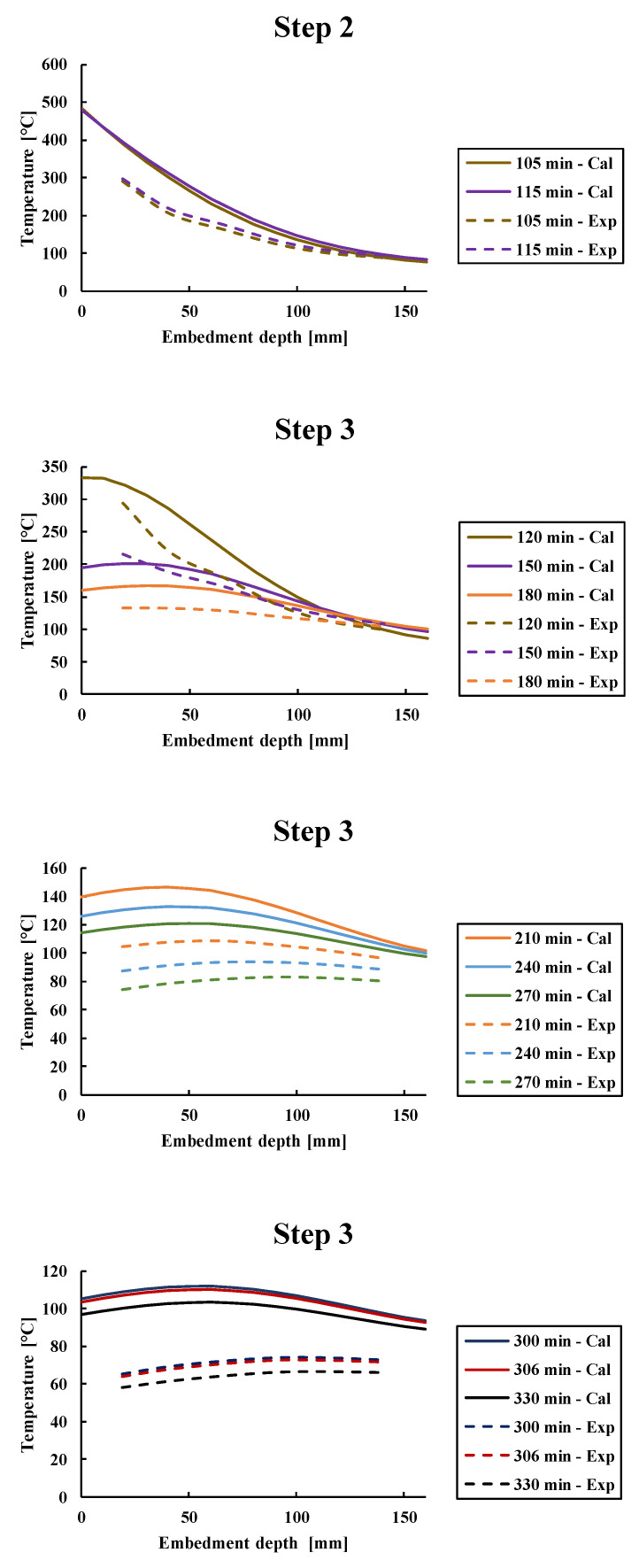
Comparison between experimentally and numerically measured temperature profiles along the rebar interface. Explanations for each step of Figure 8 were described in Section 4.1. Step 3 in Figure 8 was represented in three figures, where the temperature—embedment depth relationship was presented from 120 min to 330 min.

First, the numerical thermal simulations were compared to the results of experimentally measured temperature profiles at the steel/mortar interface at the time of fire exposure followed by the post-fire phase. Figure 8 shows the comparison between the numerically calculated (continuous lines) via the transient thermal analysis and experimental (dashed lines) temperature profiles considering the full set of thermocouples between 19 mm and 140 mm depth. Results have shown that temperature profiles obtained numerically were higher than experimental temperature profiles. The difference between experimental and numerical temperature profiles is due to the consideration of an insulating material in the calculation model, with thermophysical properties that are safer than the real thermophysical properties of the insulation used during the tests. The second reason is the use of Eurocode conservative fire conditions which are represented at a homogeneous close distance from the exposed surface [11]. Nevertheless, one could observe that despite the differences between experimental and predicted numerical temperature profiles obtained by using Eurocode thermophysical properties, the model yields conservative results, which assure a safe use of the model for design purposes. In order to be used as a predictive tool, the true thermophysical parameters of mortars and insulating material should be in the numerical model instead of reglementary coefficients of Table 3 issued by Eurocodes. Better agreement between simulated and experimental temperature profiles was observed at deeper segments of the embedment where heat transfer occurs via conduction only. However, for both experimental and numerical calculations, steep thermal gradients along the PIRs were observed near the heated surface of concrete beams, while thermal gradients were lower in the inner depths of the specimens. Furthermore, it is shown that quasi-constant temperature profiles in the post-fire phase were observed for the different cooling times after exposure to fire conditions.

**Figure 9:**
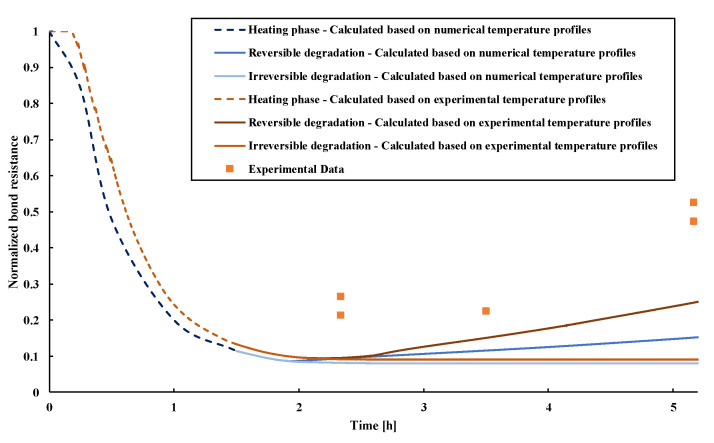
Calculated and experimental normalized post-fire pull-out resistances of 16 mm PIR with 155 mm ± 3 mm embedment depth.

To calculate the evolution of the pull-out capacity of PIRs subjected to temperature variation, Pinoteau’s method was used in the calculated bond resistances in Figure 9. Hence, the resistance profiles are estimated during both heating and cooling phases by using the experimental temperature distribution recorded during the tests, and computed temperature profiles obtained by the transient thermal analysis. The calculated post-fire pull-out resistances were considered by assuming both hypotheses: an irreversible and reversible bond strength degradation during the cooling phase. The used bond stress vs. temperature relationship available only during the heating phase for this mortar was used. At the section points where no experimental values of temperature were available, temperatures were extrapolated through the depth by a temperature vs. depth curve. Figure 9 shows a comparison between post-fire tests and the outcome of Pinoteau’s method for the epoxy-based mortar used in this study. The dashed lines correspond to the calculated resistances during the heating phase up to 90 min, while the continuous lines refer to the calculated resistances during the post-fire phase. It should be noted that the bond resistances continue to decrease in the first minutes of the cooling phase (right after the end of the heating phase) due to the continuous heat diffusion to the cooler parts toward the unexposed surface. It is shown that experimental post-fire pull-out resistances are higher than the calculated resistances. The numerically obtained curve based on 3D analysis yielded conservative results compared to the experimentally obtained results. This result indicates that such curves can be the basis of a post-fire design model of PIRs based on conservative assumptions.

Therefore, for this mortar type and test configuration, Pinoteau’s method yields conservative pull-out calculations of PIRs during the cooling phase, which justifies the need to characterize more precisely the bond resistance vs. temperature relationship of PIRs during the cooling phase for more accurate results.

## 5. Conclusions

This study aimed at providing results and preliminary information on the evolution of the pull-out capacity of PIRs made with polymeric mortars during the post-fire phase after exposure to ISO 834-1 standard fire conditions. The experimental results showed that post-fire pull-out resistance increases with cooling time (23% and more than 50% of the resistance at ambient temperature were respectively obtained at the lowest and highest cooling time). This conclusion can be associated to a probable strength recovery of the epoxy-based mortar during the cooling phase. This paper presents a validation of a numerical model for calculating the temperature profiles along the embedment depth of the PIR via a transient heat transfer analysis, after exposure to ISO 834 fire followed by cooling to ambient temperature. The temperature profiles were then used as input data for Pinoteau’s RIM used for calculating the post-fire pull-out capacity of PIRs in concrete. Pinoteau’s method includes the bond stress vs. temperature relationship of the considered mortar. In this study, the model was validated with experimental results obtained in previous experimental study [14]. For the considered mortar, it results in conservative calculations of post-fire load capacities at various fire exposure times followed by cooling compared to experimental results.

The comparison between test results and calculated results using Pinoteau’s method showed that the method underestimates the pull-out capacity of PIRs during the cooling phase for the case of this mortar type with an embedment depth of 155 mm ± 3 mm. The variation of temperature profiles during the cooling phase was confirmed to have a significant influence on the post-fire resistance of PIRs. To develop a more rational characterization of the bond resistance vs. temperature relationship of PIRs during the cooling phase as basis for safe and reliable post-fire design concept, more investigation is needed. 

This study can be pursued in the future by conducting a sensitivity analysis on radiative and convective heat transfer coefficients and emissivity for exposed and unexposed surfaces of concrete and steel to use the proposed model for prediction. It can also be pursued by performing a full coupled thermal-mechanical analysis.

## Figures and Tables

**Table 1:** Composition of concrete used for casting of beams.

Designation	Mineralogical Nature	Quantities (kg/m^3^)
G 0/6.3 Sand	Silico-Limestone	880
G 4/14 Gravel	Silico-Calcareous	792
G 4/22.5 Gravel	Silico-Calcareous	88
CEM II/B-LL 32.5R CE CP2 NF	-	320
Water	-	227

**Table 2:** Cooling duration after fire exposure.

Specimen	Cooling Time after Exposure to Fire Conditions
B1	50 min
B2	50 min
B3	2 h
B4	3 h 40 min
B5	3 h 40 min
B6	24 h

**Table 3:** Radiative and convective heat transfer coefficients and emissivity for exposed and unexposed surfaces of concrete and steel.

	Convective Heat Transfer Coefficient	Emissivity of Concrete and Steel
Fire exposed surface	$25\;{W\;m}^{-2}\;{}^{\circ}C$	0.7
Unexposed surface	$4\;{W\;m}^{-2}\;{}^{\circ}C$	0.7

## Data Availability

Not applicable.
